# “Do You Think You Have What it Takes?” – Exploring Predictors of Pre-Service Teachers’ Prospective ICT Use

**DOI:** 10.1007/s10758-021-09551-0

**Published:** 2021-07-15

**Authors:** Marcela Pozas, Verena Letzel

**Affiliations:** 1 Professional School of Education, Humboldt-Universität Zu Berlin, Unter den Linden 6, 10099 Berlin, Germany; 2 School of Psychology, University of Monterrey, Monterrey, Mexico; 3 Section for Teacher Education and Research, University of Trier, Trier, Germany

**Keywords:** Prospective ICT use, Attitudes, Self-efficacy, Digital competencies, Gender differences

## Abstract

Despite extensive efforts to support teachers with the integration of information and communication technologies (ICT) into their classroom practice, current research reports that teachers face immense challenges when integrating ICT into their teaching. This issue has become even more relevant with the rapid spread of the COVID-19 virus, forcing schools around the world to close for an indefinite period of time and thus to offer remote digital learning solutions. Against this background, this study focused on examining the predictors of pre-service teachers’ prospective ICT use and investigated the heterogeneous results of previous research related to ICT use and gender. Following the ‘will, skill, tool’ framework, the study examined relevant factors of pre-service teachers’ (*N* = 103) prospective ICT use for teaching and learning processes by means of multiple regression analyses. The analyses included pre-service teachers’ background characteristics, ICT profiles (attitudes and self-efficacy), digital competencies and use of digital tools in order to explore their role in future in-class use of ICT. They also show that there are no gender differences in pre-service teachers’ prospective ICT integration. However, male pre-service teachers hold more positive attitudes towards ICT use than their female counterparts. Additionally, the findings reveal that the two strongest predictors of pre-service teachers’ future ICT use are their attitudes and perceived competency to teach and implement technology in their teaching practices. Finally, the results provide important information about teachers’ training needs. Implications of the results and further research are discussed.

## Introduction

The pedagogically sound integration of information and communication technology (ICT) can support learning processes and improve the quality of education (Drossel et al. 2016; Eickelmann 2011; Pacurar & Abbas, 2015), depending on the way in which it is implemented in classrooms (Seufert et al. 2021). Hence, teachers play a crucial role in the effective incorporation of ICT into classrooms (Davis et al. 2013; Fraillon et al. 2019). Research has shown that teachers’ ability to use ICT can enhance students’ learning (OECD 2010), foster students’ motivation (Rodrigues 2010) and promote students’ ICT use (UNESCO 2011). Consequently, research and scientific literature have focused on the factors determining and hindering ICT integration by both pre-service teachers, i.e. students currently enrolled in teacher training programmes leading to initial certification and in-service teachers, i.e. teachers who have entered the teaching profession (Eickelmann 2011; Hämäläinen et al. 2021; Tondeur, Petko et al. 2020). Among these factors, attitudes, self-efficacy, ICT experience and ICT competencies have been identified as significant and relevant elements related to ICT use (Drossel et al. 2016; Knezek & Christensen, 2016; Grafe & Breiter, 2014; Rubach & Lazarides, 2019; Spiteri & Rundgren, 2020). However, even though considerable efforts have been made to identify the determinants of and barriers to ICT integration and thus to support teachers with integrating ICT into their classroom practice (Baturay et al. 2017; OCED 2014), the literature shows that teachers still infrequently use ICT (Tondeur et al. 2013; Uerz et al. 2018). Furthermore, it has also been reported that teachers make use of ICT mainly for administrative purposes, rather than for actual teaching (Krause et al. 2017). Additionally, pre-service teachers report feeling insufficiently prepared to effectively integrate ICT into their teaching (Tondeur et al. 2017).

Overall, in-service and pre-service teachers have struggled to integrate ICT into teaching and learning processes in recent years. This ongoing challenge has become even more pressing with the current global COVID-19 health crisis. With the rapid spread of COVID-19 forcing schools around the world to close, emergency remote education was adopted, shifting face-to-face instruction to online learning (Bozkurt & Sharma, 2020; Seufert et al. 2021). This hasty transition revealed obstacles hindering ICT integration into the educational landscape. In order to generate findings on digitalization and schooling in times of the COVID-19 crisis, Schuknecht and Schleicher (2020) investigated the digital infrastructure of the OECD countries and their teachers’ technical and pedagogical skills. Their results indicate that there is still a considerable need to invest in infrastructure and to improve teachers’ ICT competencies and the learning environment. In line with this study, recent research conducted in Portugal further revealed that successful implementation of emergency remote education was severely limited because teachers lacked training, competencies and technical equipment (Sá & Serpa, 2020). Indeed, recent reports have revealed that teachers face significant challenges and rarely make use of digital tools or online courses. For example, a study by König et al. (2020) conducted in Germany reported that the majority of teachers did not provide online lessons, with almost 70% of teachers not using any digital instruments to teach online. Only 20% of them conducted online assessment. Further analyses by König et al. (2020) showed that, as expected, teachers’ digital competencies were related to how they mastered the challenges during emergency remote education.

With this background and given the current worldwide situation, the question of whether pre-service teachers are prepared to face the current challenges in the digital educational landscape has become more relevant. Shockingly, various recent studies have revealed that ICT plays a minor role in teacher training programmes (Tiede 2020; Tiede et al. 2015). Indeed, pre-service teachers report that ICT-related content is not discussed in-depth in their teacher training programmes (Jäger-Biela et al. 2020) and that they do not feel prepared to effectively integrate ICT into their teaching practices (Gill et al. 2015; Tondeur et al. 2017). Consequently, there is still no clear picture of pre-service teachers’ digital competencies (Rubach & Lazarides, 2019).

Against this backdrop, it is meaningful and necessary to explore pre-service teachers’ digital competencies and examine their relation to prospective ICT use in order to identify specific factors that inform teacher training. Given that pre-service teacher training plays a crucial role in promoting the integration of ICT into classrooms (Botturi 2019), Tiede (2020) strongly underlines the importance of deriving content for teacher training curricula from relevant scientific literature and research in order to ensure that pre-service teachers are specifically prepared for their prospective teaching profession. To achieve these goals, this study acknowledges the importance of following appropriate conceptual models of educational technology integration (Tondeur et al. 2020). In this vein, the study was framed within the ‘will, skill, tool’ model by Knezek and Christensen (2016), which has been used previously by stakeholders and decision makers as a means of determining factors that need to be addressed in professional development and training programmes (Tondeur et al. 2020). Additionally, because prior research has yielded heterogeneous evidence concerning the impact of gender on ICT-related topics (e.g. attitudes, ICT use), the study investigated potential gender effects (Krause et al. 2017).

The next sections are organized as follows. Section 2 will introduce the conceptual model ‘will, skill, tool’ (WST), followed by a description of the relevant literature and research related to each of the three constructs of the WST. Section 3 will discuss findings from ICT research related to the impact of gender.

## The ‘will, skill, tool’ model

The ‘will, skill, tool’ (WST) model is a well-established theoretical framework which contains all the necessary components for the successful integration of ICT into teaching and learning processes (Christensen & Knezek, 2008). The model consists of three core elements (Knezek & Christensen, 2016): (a) ‘will’, which constitutes a positive attitude towards ICT integration into education; (b) ‘skill’, which denotes the ability and self-efficacy to use ICT and (c) ‘tool’, which refers to the availability and extent of use of ICT. The WST model has a great range of flexibility, as each of the three constructs can be assessed using different measurement instruments (Tondeur et al. 2020). Moreover, according to Tondeur et al. (2020), the WST is consistent with other technology integration models such as the Technological Pedagogical Content Knowledge (TPACK) framework and is broad in scope, as it has been used in different countries (Agyei & Voogt, 2010; Farjon et al. 2019).

Previous research has shown that these three constructs explain a very high degree of variance in the frequency of classroom ICT use. Knezek et al. (2003) reported that measures based on this model accounted for 64–83% of variance in ICT integration by teachers. Similarly, Morales (2006) conducted a transnational study of the model with teachers from Mexico and the United States and reported that the WST model accounted for 90–96% of variance of ICT use across the sample. A more recent study by Petko (2012) indicated that 60% of variance could be explained by factors from the model. A few studies have also used the WST model to investigate pre-service teachers’ use of ICT. Agyei and Voogt (2010), for example, compared pre-service and in-service mathematics teachers’ ICT use in Ghana. A more recent study by Farjon et al. (2019) revealed that the WST model explained 60% of pre-service teachers’ technology integration. Additionally, their findings indicated that pre-service teachers’ attitudes were the strongest predictor of pre-service teachers’ technology integration in the model.

### Pre-Service Teachers’ ICT Profiles: Attitudes (will)

Ajzen (2005) defined attitudes as dispositions to respond favourably or unfavourably towards an object, person or event. Attitudes can be inferred from an affective, behavioural and cognitive component response towards the object of a specific attitude (Ajzen, 2005). They are considered to be a determinant of behavioural intention (Ajzen 1991). As they are strongly related to actions (Haddock & Maio, 2014), they play a considerable role in teachers’ classroom practices (Baumert & Kunter, 2006; Schaarschmidt 2005). In this context, many studies have focused on measuring the impact of attitudes on ICT integration. Within the WST model, attitudes are considered to be a strong predictor of in-class ICT integration (Knezek & Christensen, 2016). Indeed, empirical evidence shows that favourable attitudes towards ICT influence teachers’ technology integration (Knezek & Christensen, 2016; Petko 2012; Seufert et al. 2021). Likewise, research has found that attitudes towards ICT have a strong positive relation with the intention to use ICT in class (Celik & Yesilyurt, 2013; Kreijn et al. 2013; Petko 2012; Sang et al. 2010; Scherer & Teo, 2019). Additionally, Bas et al. (2016) argue that in-service teachers who hold less positive attitudes towards ICT commonly invest less effort into adopting ICT in their instructional practices. A more recent study by Gretter and Yadav (2018) indicateS that pre-service teachers generally hold positive attitudes towards ICT and thus consider the use of ICT for educational purposes to be of great importance.

### Self-Efficacy and Digital Competencies (skill)

The *skill* construct within the WST model represents an individual’s self-efficacy, ability and experience related to the use of ICT (Baturay et al. 2017; Tondeur et al. 2020). Woolfolk (2004) defines self-efficacy as an individual’s belief in his or her ability to manage and handle situations. Self-efficacy has been positively related to an individual showing effective coping behaviours when faced with computer-related difficulties. Recent evidence shows that teachers who perceive higher levels of self-efficacy experience less ICT-related anxiety and stress (Dong et al. 2020). Previous research has indicated that teachers’ self-efficacy towards ICT use plays an important role in how they integrate ICT into their instruction (Gil-Flores et al. 2017). Moreover, according to Sang et al. (2010), pre-service teachers’ self-efficacy predicts their prospective computer use in education.

Teachers’ digital competencies are also an important determinant of ICT use in education (Tiede et al. 2015). Worldwide, several well-established ICT competency frameworks have been proposed. Different terms such as ICT literacy, digital literacy and ICT competency are used in these frameworks (Markauskaite 2006). According to Tondeur et al. (2017), the term ICT competency is most widely used as it encompasses a more comprehensive view of ICT use. In this context, digital competency can be defined as the functional use of digital knowledge, skills and attitudes (Ananiadou & Claro, 2009).

Institutional bodies regulating teacher training standards worldwide (e.g. in the United States of America and Germany) have incorporated ICT competencies into their guidelines (e.g. the International Society for Technology in Education, 2008, in the United States of America; the Standing Conference of the Ministers of Education and Culture, KMK, 2016, in Germany). In Germany, the country where this study was conducted, the need to prepare pre-service teachers has been long acknowledged. In line with the European Digital Competence Framework (DigComp) (Ferrari 2013), the KMK released a strategy paper comprising the six digital competencies (searching and organizing, communicating and cooperating, producing and presenting, protecting and acting safely, problem-solving and handling, analysing and reflecting) that teachers are expected to foster in their students across subjects. To achieve this task, teachers and pre-service teachers (Rubach & Lazarides, 2019) need to acquire such competencies themselves. Thus, ICT-related contents are obligatorily integrated into teacher training curricula (Tiede 2020). However, in order to acquire digital pedagogical competencies, pre-service teachers can either choose elective courses during their teacher training programmes, obtain additional certificates on top of their teacher training certification or complete graduate studies focusing on specific aspects of digital pedagogy (Tiede et al. 2015). Therefore, it is not surprising that pre-service teachers still feel unprepared to effectively integrate ICT into their teaching practices (Gill et al. 2015; Tondeur et al. 2017). In addition, research has indicated that the instructors of teacher training courses report a lack of experience in ICT for educational purposes and, therefore, feel unprepared to support and train pre-service teachers (Barbour & Harrison, 2016; Urez et al. 2018).

### ICT use (tool)

Another important predictor of ICT integration is teachers’ previous experience and use of ICT. According to Christensen and Knezek (2008), teachers who use ICT integrate instructional ICT tools into their teaching practice more frequently than colleagues who have less ICT experience. Results from the International Computer and Information Literacy Study (ICILS) 2013 revealed that teachers’ experience in ICT for educational purposes is moderately associated with their frequency of ICT use (Fraillon et al. 2014). According to Breiter et al. (2010), the use of a wide range of media in the classroom positively impacts teaching practice. Furthermore, Petko (2012) argues that teachers who constantly use ICT for private and professional purposes tend to include ICT more often in their teaching practice. Rubach and Lazarides (2019) followed this line of thought and explored the relationship between pre-service teachers’ self-reported digital competencies and the diversity of use of digital resources and tools. They suggested that the use of a wide range of digital tools could have an impact on pre-service and in-service teachers’ behaviour in the classroom.

## Gender Differences and ICT

Previous research has revealed that personal factors are associated with ICT-related constructs and use. One of the most intensively investigated personal factors is gender (Padilla-Meléndez et al. 2013; Sang et al. 2010). However, the topic of gender differences appears to be complex, as empirical research has yielded mixed evidence on the impact of gender on ICT-related constructs (Hatlevik and Arnseth 2012; Siddiq et al. 2015). With respect to ICT self-efficacy, the ICILS reported that male teachers showed higher ICT self-efficacy than female teachers (Gebhardt et al. 2019). Additionally, a higher percentage of female than male teachers reported using ICT in their instruction. Moreover, female and male teachers did not differ in general in their attitudes towards ICT. However, gender differences were observed in some countries such as Croatia and Poland. Similarly, evidence from an international comparison by Drossel et al. (2016) revealed country-specific gender differences. For example, female teachers in the Netherlands reported a higher frequency of ICT use for educational purposes than male teachers. In Poland and Germany, on the other hand, male teachers reported more frequent ICT use in the classroom than female teachers. In contrast, the findings from a study by Hatlevik and Hatlevik (2018) did not reveal any significant associations between teachers’ gender and ICT self-efficacy or even ICT use for pedagogical purposes. In light of these results, pre-service teachers’ gender seems to be a relevant personal factor for the analyses in this study.

## Research aims

The current health crisis has placed the spotlight on ICT integration, a topic that has been on the education agenda for years. COVID-19 forced schools around the world to close, requiring teachers to shift quickly to emergency remote online education. However, despite previous efforts, the development of pre-service and in-service teachers’ digital competencies and technology use has remained critical. Additionally, there is still a lack of research on the assessment of pre- and in-service teachers’ ICT competencies (Rubach & Lazarides, 2019, 2021). Against this background and framed by the WST model (Knezek & Christensen, 2016), this study had the primary goal of investigating pre-service teachers’ digital competencies and also explored the predictors of their prospective ICT use. Additionally, given the heterogeneous evidence on gender differences, the study investigated potential gender effects on pre-service teachers’ ICT profiles (attitudes and self-efficacy), digital competencies and use of digital tools. The research questions guiding the study were:

1. How digitally competent do pre-service teachers perceive themselves to be?
2. Do the ICT profiles (attitudes towards ICT use, self-efficacy towards ICT use), digital competencies and use of digital tools of male and female pre-service teachers differ?
3. How do pre-service teachers’ ICT profiles (attitudes towards ICT use, self-efficacy towards ICT use), digital competencies and use of digital tools influence their prospective computer use?

## Method

### Participants and Procedure

Data were collected from 103 teacher education students at a public university in Germany. Only students studying to become secondary school teachers participated in the study. The mean age of the sample was 25.10 years (SD = 3.66 years). Of the 103 participants, 76% were female. Most of the students were doing Master’s studies (83%) and about 18% were doing Bachelor’s studies. All data were collected during the 2020 summer semester during the COVID-19 pandemic. To recruit participants, an online survey was sent to pre-service teachers, i.e. students attending teacher education lectures and courses. Participation was voluntary and the questionnaires were completed anonymously.

### Instruments

#### Demographic Information

Firstly, demographic information was collected. This included information about gender (dummy coded: male = 1; female = 2), age, the number of semesters in the teacher education programme, the school track programme and school internships. The participants were also asked to indicate whether they had already attended seminars or courses on the use of digital media in school during their studies.

#### Attitudes Towards ICT use

Pre-service teachers’ attitudes towards ICT use were measured with a scale based on the work of Tappe (2017) and Nistor et al. (2012). The scale consisted of four items (e.g. ‘I am happy when I can make use of digital teaching elements in my classes’ (α = 0.88); a 4-point scale was used to rank the items (1 = *does not apply* to 4 = *applies completely*).

#### Self-Efficacy Towards ICT use

To assess pre-service teachers’ self-efficacy towards ICT use, a scale by Tappe (2017) was used. It comprised five items (e.g. ‘I can plan a lesson including digital teaching elements even if I have only a limited amount of time to use them’). The scale consisted of a 4-point scale ranging from 1 (*does not apply*) to 4 (*applies completely*). The internal consistency of the scale for the present sample was α = 0.86.

#### Pre-Service Teachers’ Self-Reported Digital Competencies

Pre-service teachers’ self-reported digital competencies were measured using an instrument by Rubach and Lazarides (2019). Based on the ‘will, skill, tool’ model (Knezek & Christensen, 2016), this model assesses pre-service teachers’ self-reported beliefs about their digital competencies and integrates the competencies set out by the German Standing Conference of the Ministers of Education and Cultural Affairs’ (KMK) in ‘Education in the Digital World^1^’. The instrument consists of seven scales: searching and organizing (e.g. ‘I can analyse, interpret and critically evaluate information and files; α = 0.55), communicating and cooperating (e.g. ‘I can share information, files and links’; α = 0.53), producing and presenting (e.g. ‘I can edit, join, present, publish and share content in different formats’; α = 0.68), protecting and acting safely (e.g. ‘I know the dangers and risks of the digital environment and I therefore consider and reflect on them’; α = 0.62), problem-solving and handling (e.g. ‘I can identify digital opportunities for learning and thus identify, evaluate and use the appropriate tools’; α = 0.83), analysing and reflecting (e.g. ‘I know how diverse the media landscape is’; α = 80) and teaching and implementing (e.g. ‘I can identify the potential of integrating digital media into teaching’; α = 0.81). The instrument has a 5-point scale (1 = *does not apply at all* to 5 = *applies completely*). Given that the internal consistencies were low for the scales ‘searching and organizing’ and ‘communicating and cooperating’, we decided to exclude these scales from further analyses.

#### Use of Digital Tools

Students were given a list of 26 digital devices, resources and tools (e.g. computer, WhatsApp, blogs). Using a dichotomous response format (yes/no), they specified the digital instruments they used for private or study purposes. Based on their responses, we calculated two sum scores comprising all the digital instruments used for (a) private and (b) study purposes.

#### Prospective Computer use for Educational Purposes

Pre-service teachers’ prospective computer use for educational purposes was measured using an adapted version of the *Prospective Computer Use Scale* by Sang et al. (2010). Applying a combined technique, we translated the scale into the German language (Cha et al. 2007). The scale consists of 10 items based on a 3-point Likert scale ranging from 1 (*not at all interested*) to 3 (*very interested*) (e.g. ‘I would use the computer to assist with the differentiation or implementation of individual learning plans’; α = 0.87).

## Results

### Pre-Service Teachers’ ICT Profiles and Digital Competencies

Mean and standard deviation scores were calculated to determine the pre-service teachers’ attitudes, self-efficacy, digital competencies and ICT use. As shown in Table 1, the values for pre-service teachers’ attitudes, self-efficacy towards ICT use and digital competency were between 2.68 to 4.28. As the theoretical mean of the scales was 2.5, the scores were significantly positive: total mean scores for attitudes towards ICT use (*t*(102) = 6.98, *p* < 0.01), producing and presenting (*t*(102) = 16.25, *p* < 0.01), protecting and acting safely (*t*(102) = 13.43, *p* < 0.01), problem-solving and handling (*t*(102) = 8.78, *p* < 0.01), analysing and reflecting (*t*(102) = 13.08, *p* < 0.01) as well as teaching and implementing (*t*(102) = 13.09, *p* < 0.01). However, the scores for pre-service teachers’ self-efficacy towards ICT use were significantly lower, (*t*(102) = -3.84, *p* < 0.01).

**Table 1:** Means, standard deviations and correlations of all variables

	*M*	*SD*	1	2	3	4	5	6	7	8	9	10	11	12
Attitudes towards ICT use	3.61	.89	–											
Self-efficacy towards ICT use	2.68	.85	.48**	–										
Producing and presenting	4.28	.80	.26**	.27**	–									
Protecting and acting safely	3.97	.73	.23*	.25**	.32**	–								
Problem-solving and handling	3.73	.84	.47**	.39**	.43**	.49**	–							
Analyzing and reflecting	3.99	.77	.45**	.32**	.27**	.45**	.62**	–						
Teaching and implementing	3.99	.76	.47**	.31**	.24**	.25*	.54**	.64**	–					
ICT use for private purposes	12.20	2.82	.40**	.18	.31**	.08	.43**	.32**	.22*	–				
ICT use for study purposes	9.84	3.39	.35**	.14	.36**	.20*	.32**	.22*	.12	.24*	–			
Prospective computer use for educational purposes	2.04	.47	.75**	.29**	.20*	.14	.42**	.38**	.52**	37**	.32**	–		
Age	25.09	3.66	-.03	− .15	− .11	− .08	− .06	.03	− .07	− .26**	− .23**	.08	–	
Gender	–	–	− .24*	− .18	.06	.06	− .11	− .14	− .02	− .17	− .09	− .16	− .08	–

Concerning the pre-service teachers’ use of ICT tools, the total mean score for ICT use for study purposes (t(102) = − 7.99, p < 0.01) was significantly lower, as the theoretical mean of the scales was 12.5. No significant difference to the theoretical mean was found for students’ ICT use for private purposes. The total mean score for pre-service teachers’ prospective ICT use (theoretical mean: 2) was significantly positive, (*t*(102) = 8.79, *p* < 0.01).

### Gender Differences in Pre-Service Teachers’ ICT Profiles and Digital Competencies

Correlations were calculated among all variables and gender. As shown in Table 1, a weak but negatively significant correlation was found between pre-service teachers’ attitudes towards ICT and gender, indicating that female pre-service teachers hold less positive attitudes towards ICT use. No other significant correlations were found for the rest of the variables under study. Given that more than 70% of the respondents were female, we decided to explore this correlation using the Mann-Witney nonparametric test (Field 2013). In line with the correlation analysis, the results show that the attitudes towards ICT of male pre-service teachers (*Mdn* = 4.00) are more positive than those of female pre-service teachers (*Mdn* = 3.75), *U* = 640.50*, z* = − 2.59*, r* = − 0.26.

### Predictors of Pre-Service Teachers’ Prospective ICT use

To explore the predictors of pre-service teachers’ prospective ICT use, we calculated a multiple regression analysis. Prospective computer use for educational purposes was included as the dependent variable as a measure of pre-service teachers’ future use of ICT in their teaching practice. Following the WST framework, a four-stage multiple regression analysis was performed. Model 1 controlled for the sociodemographic variables of age and gender. Model 2 introduced the ‘will’ predictor of attitudes towards ICT use; Model 3 included the ‘skill’ predictors of self-efficacy towards ICT use and pre-service teachers’ digital competencies. Finally, Model 4 added the ‘tool’ predictors of ICT use for private and study purposes to the regression equation. The results are displayed in Table 2. The VIF values from all the multiple regression models revealed that multicollinearity was low (< 2). Autocorrelation was revised using the Durbin-Watson statistic which indicated an acceptable level of 2.10 (Savin and White 1978).

**Table 2:** Multiple regression models: prediction of preservice teachers’ prospective computer use based on the WST framework

Variable	Model 1	Model 2	Model 3	Model 4
	*β*	*β*	*β*	*β*
Age	.07	.11	.11	.13
Gender	− .13	.04	.002	.01
*Will*: Attitudes towards ICT use		.76**	.69**	.68**
*Skill*: Self− efficacy towards ICT use			− .01	− .07
*Skill*: Producing and presenting			.00	− .02
*Skill*: Protecting and acting safely			− .04	− .02
*Skill*: Problem− solving and handling			.07	.05
*Skill*: Analysing and reflecting			− .11	− .13
*Skill*: Teaching and implementing			.28**	.28**
*Tool*: ICT use for private purposes				.05
*Tool*: ICT use for study purposes				.02
*R*^2^	.02	.57	.63	.63
*ΔR*^*2*^	.02	.54**	.06*	.00

+ *p* < .10; *p < .05; **p < .01

The multiple regression analyses revealed that the inclusion of age and gender as covariates in the regression equation explained only an insignificant 2% of pre-service teachers’ prospective ICT use (Model 1). Attitudes towards ICT use, on the other hand, were revealed to be a powerful predictor of pre-service teachers’ prospective ICT use, (*F*(3, 101) = 43.29, *p* < 0.001), accounting for 57% of the variation of their prospective ICT use. The addition of self-efficacy and pre-service teachers’ digital competencies to the model explained an additional 6% of variation, augmenting the models R^2^ significantly, (*F*(9, 101) = 17.01, *p* < 0.001). Finally, when both measures of ICT use for either private or study use were controlled (Model 4), all variables accounted for 63% of the variance, (*F*(11, 101) = 13.79, *p* < 0.001). The results indicate that when pre-service teachers hold more positive attitudes towards ICT use and perceive themselves as competent in teaching and implementing ICT resources for educational purposes, they are more likely to make use of technology in their future instructional practice.

## Discussion

This study investigated pre-service teachers’ digital competencies and strove to determine the predictors of their prospective ICT use. The mean and standard deviation scores of pre-service teachers’ ratings suggest that their self-perceived competence in all digital skills is fairly positive. In detail, the competency of ‘producing and presenting’ was rated especially positively by the sample. This indicates that pre-service teachers feel confident that they know a broad variety of digital programs and apps and that they are able to present content in different digital formats. However, it is important to highlight that such data do not provide information on the type of ICT resources pre-service teachers use for producing and presenting information. That is, we do not know whether they use special ICT resources for instructional purposes or normal data processing software such as Microsoft Office. In line with previous studies on pre-service teachers’ attitudes towards ICT (Gretter & Yadav, 2018), the sample in this study seemed to have positive attitudes towards ICT. Likewise, pre-service teachers’ ratings of their intended prospective ICT use were rather positive. Interestingly, self-efficacy towards ICT use was rated as rather negative by the sample. Although this result is consistent with previous research indicating that pre-service teachers perceive themselves as less self-efficacious with regard to ICT use for instructional purposes (Valtonen et al. 2021), it is surprising that they feel fairly positive about their competency in ‘teaching and implementing’. Such a competency comprises the use of ICT resources for in-class teaching. Nonetheless, based on studies of other pre-service teacher samples, Rubach and Lazarides (2019, 2020) also report that such a competency was rated fairly positively. Maderick et al. (2015) argue that pre-service teachers might consider themselves ‘competent’ based upon the “extensive yet extremely narrow spectrum of recreational use of digital technologies versus competence regarding actual professional requirements” (p. 3). However, prior research has also demonstrated that familiarity with ICT resources and tools does not necessarily translate into pre-service teachers’ use of technology for instructional purposes (Gretter & Yadav, 2018). Thus, this study’s results could perhaps be explained by the fact that pre-service teachers overestimate their competence, given their familiarity with and high usage of ICT resources for private purposes.

The Pearson’s correlation revealed no significant relationships between gender and the variables of pre-service teachers’ digital competencies, self-efficacy, ICT for private and study use as well as prospective ICT use. These results are consistent with the findings of Hatlevik and Hatlevik (2018), Sang et al. (2010) as well as Guillén-Gámez et al. (2019). Nonetheless, we found a significant association between gender and attitudes towards ICT use. It appears that female pre-service teachers hold less positive attitudes towards ICT use than their male counterparts. Given the unbalanced sample size, we carried out nonparametric testing to confirm this result. Similar findings were also reported in a recent study by Tondeur et al. (2016) which revealed that, in general terms, females hold less positive attitudes towards ICT than males. In addition, a recently published meta-analysis on gender and attitudes towards ICT found a small but significant positive effect, suggesting that male participants hold more favourable attitudes (Cai et al. 2016). In contrast, a study by Gebhardt et al. (2019) found gender differences only in country-level analyses. Thus, it would be meaningful to explore whether this holds true for the German pre-service teacher sample in comparison to other countries. According to Ferreira (2017) and Tømte (2008), empirical research in the field of ICT suggests that female participants are falling behind in terms of a wide range of variables (e.g. attitudes, self-confidence, interest and digital skills). However, Meelissen and Drent (2008) argue that such an argument can only be considered valid if male participants’ ratings are used as the norm and, thus, as representative of the “expected” standard. Consequently, Ferreira (2017) argues that these differences might stem from deep-rooted gender stereotypes and preconceived ideas of how women and men use (or should use) technology. Hence, the statistically significant gender difference within this study does not automatically imply that female participants are falling behind the male sample. A closer look at the study’s results reveals the total mean score for the participants’ attitudes towards ICT use was significantly higher than the mean (c.f. Section 6.1), indicating that in general both male and female participants hold positive attitudes towards ICT. More specifically, in this sample, only 3% of the female participants can be considered to be at risk (given their extremely low ratings regarding their attitudes towards ICT use). Taken altogether, this study calls for critical reflection on gender differences and ICT and on the evaluation of such differences. In this vein, teacher education should focus on helping pre-service teachers to use ICT meaningfully and productively, regardless of their gender, with the goal of fostering positive attitudes towards ICT use in teaching practice.

In line with previous research, the results from the regression analysis reveal that pre-service teachers’ attitudes towards ICT use was the strongest predictor of their prospective ICT use in classroom practices (Krause et al. 2017; Seufert et al. 2021). As discussed above, attitudes are considered a strong determinant of teachers’ behavioural intention to change their classroom practices (Baumert & Kunter, 2006; Schaarschmidt 2005). The second strongest predictor of pre-service teachers’ prospective ICT use was the competency of ‘teaching and implementing’. Although this construct comprises pre-service teachers’ self-assessed competency to make use of ICT resources for actual teaching, the items’ wordings focused on the potential of ICT use for, e.g., teaching subject content (Rubach & Lazarides, 2019). Research has highlighted that, in addition to positive attitudes, acknowledging the importance, value and potential of ICT for teaching has an impact on ICT integration (Gretter & Yadav, 2018). Surprisingly, pre-service teachers’ self-efficacy was not shown to be a significant predictor of their prospective ICT use. In this respect, this study’s results are not in line with the findings of previous studies (Baturay et al. 2017; Gil-Flores et al. 2017; Tondeur et al. 2020). However, as the study’s results are based on a small sample and on cross-sectional data, they should be interpreted with care. Consequently, we recommend replicating the study’s design with a bigger sample as well as using a longitudinal or cross-lagged panel design with several measurement points. Finally, the WST model explained 63% of the variation in pre-service teachers’ prospective ICT use. This finding is consistent with evidence from previous studies using the WST to investigate the determinants of both pre- and in-service teachers’ ICT integration (Farjon et al. 2019; Petko 2012).

Taking the findings from this study together, it is possible to identify specific areas that should be targeted in teacher training programmes: 1) fostering of pre-service teachers’ self-efficacy and attitudes and 2) training of pre-service teachers to use ICT as a teaching tool (Agyei & Voogt, 2010; Sang et al. 2010; Tondeur et al. 2012; Urez et al. 2018). How can this be implemented in teacher training programmes in practical terms? First, on a policy level, teacher training institutions must include education in the use of ICT for teaching purposes as core and obligatory content within their course offer. Additionally, there must be a broad offer of courses encompassing not only general pedagogical and technological contents, but also incorporating subject-specific topics. Second, pre-service teachers should be provided with experience in applying technology within their specific subject domains. In addition, they should be able to observe ICT integration during their teaching internships, practise using ICT and collaborate with their peers in authentic scenarios (Tondeur et al. 2012). To this end, teacher training institutions should develop action-oriented courses (Rubach & Lazarides, 2020) which not only provide pre-service teachers with up-to-date knowledge on the effective use of ICT instruments and tools, but also allow them to put their knowledge into practice. Pre-service teachers should also be encouraged to design lesson plans incorporating ICT as a teaching instrument, to pilot these ICT-based lesson plans and to implement them in the classroom. Third, following Hobbs et al. (2011), schools and universities should partner up to enable pre-service teachers to work together with in-service teachers in real ‘teaching’ situations (Tondeur et al. 2018). Lastly, teacher educators should be well-prepared and competent in teaching and modelling technology use. Teacher educators play a crucial role, as they serve as role models and must possess the pedagogical knowledge and skills that they want their students to acquire (Urez et al. 2018). In other words, teacher educators should not only deliver content, but should also teach and model technology use. As shown by previous research, every effort can be effective in fostering the attitudes and self-efficacy of pre-service teachers and developing their competencies (Botturi 2019; Valtonen et al. 2021).

## Limitations and further research

It should be noted that this study has several limitations. The research methodology is based mainly on self-report measures. As suggested by Maderick et al. (2015), further research should follow a more holistic approach and include more objective measures in order to determine, in particular, digital competence. Furthermore, research should follow a longitudinal research design to capture the development of pre-service teachers’ attitudes, self-efficacy and digital competencies. Finally, to predict prospective ICT use in pre-service and in-service teacher samples, research has suggested applying structural equation modelling (Aygei and Voogt 2010; Knezek et al. 2000). However, this type of data analysis requires a larger sample size. Further limitations of this study are the small sample size and the higher percentage of female participants. However, the sample distribution is representative of the distribution of female and male students in teacher education (Stephan et al. 2019). Although nonparametric testing was used to control for the unbalanced sample size, the results should be interpreted with caution. Additionally, the study was carried out at only one university in Germany. According to Tiede (2020), the integration of ICT-related courses into teacher training programmes in Germany varies considerably from state to state. For instance, in some universities in certain states, teacher training programmes include obligatory ICT courses, whereas in other universities such courses are elective courses which students can decide to take voluntarily. As a consequence, generalization of the results to other universities or teacher training institutions is hardly possible. Lastly, given that the data analysed within this study were collected in only one country, it cannot be assumed that the results are representative for other countries. Teacher education in each country has its distinctive features. In particular, teacher training programmes are shaped by specific ICT competency models in different countries (Tiede 2020). In this context, it is of upmost importance to conduct international comparative research to provide additional insights beyond a single research background.

## Conclusion

In sum, the findings from this study underline the responsibility of teacher education institutions in conveying knowledge about how to effectively use ICT and thus allow pre-service teachers to competently implement ICT in their future teaching. Furthermore, the results of this study suggest that teacher training programmes should not only foster pre-service teachers’ competencies but also pay special attention to pre-service teachers’ attitudes and self-efficacy. Given the current crisis, it can be concluded that, now more than ever, pre-service teachers need to be provided with well-rounded ICT training based on authentic experiences, preparing them for their future role as teachers who can effectively include ICT in their daily teaching practice.

## Footnotes

1 For further information on the ‘Education in the Digital World’ strategy, please refer to the Standing Conference of the Ministers of Education and Culture (*Ständige Kultusministerkonferenz, KMK*).

## References

[CR1] Ajzen I (1991). The theory of planned behavior. Organizational Behavior and Human Decision Processes.

[CR2] Ajzen I (2005). Attitudes, Personality and Behavior.

[CR3] Ahsan M, Sharma U, Deppeler J (2012). Exploring pre-service teachers’ perceived teaching-efficacy, attitudes and concerns about inclusive education in Bangladesh. International Journal of Whole Schooling.

[CR4] Ananiadou, K. & Claro, M. (2009). 21st century skills and competences for new millennium learners in OECD countries*.* Paper presented at the *New Millennium Learners Conference, 21st Century Skills and Competencies*, Brussels Department of Education.

[CR5] Agyei DD, Voogt JM (2010). Exploring the potential of the will, skill, tool model in Ghana: Predicting prospective and practicing teachers’ use of technology. Computers & Education.

[CR6] Bas G, Kubiatko M, Murat A (2016). Teachers’ perceptions towards ICTs in teaching-learning process: Scale validity and reliability study. Computers in Human Behavior.

[CR7] Baran E, Bilici SC, Albaryrak A, Tondeur J (2017). Investigating the impact of teacher education strategies on preservice teachers’ TPACK. British Journal of Educational Technology.

[CR8] Barbour MK, Harrison KU (2016). Teachers’ perception of K-12 online: Impacting the design of a graduate course curriculum. Journal of Educational Technology Systems.

[CR9] Baturay M, Gökçearslan Ş, Ke F (2017). The relationship among pre-service teachers’ computer competence, attitudes towards computer-assisted education and intention of technology acceptance. International Journal of Technology Enhanced Learning.

[CR10] Bauer, P. (2011). Vermittlung von Medienkompetenz und medienpädagogischer Kompetenz in der Lehrerausbildung [ICT Competence Development in Teacher Training]. In T. Köhler & J. Neumann (Hrsg.), Wissensgemeinschaften. Digitale Medien – Öffnung und Offenheit in Forschung und Lehre (Medien in der Wissenschaft, Bd. 60, S. 294–303). Münster: Waxmann.

[CR11] Baumert J, Kunter M (2006). Stichwort: Professionelle Kompetenz von Lehrkräften [Keyword: Teachers‘ professional competence]. Zeitschrift Für Erziehungswissenschaft [journal for Educational Sciences].

[CR12] Breiter, A., Welling, S., & Stolpmann, B. E. (2010). Medienkompetenz in der Schule. Integration von Medien in den weiterführenden Schulen in Nordrhein-Westfalen [Digital competence in school. Intergration media in schools in Nordrhein Wastfalia]. *Schriftenreihe Medienforschung der LfM [Written Media Research]*. http://lfmpublikationen.lfm-nrw.de/modules/pdf_download.php? products_id=237

[CR13] Botturi L (2019). Digital and media literacy in pre-service teacher education: A case study from Switzerland. Nordic Journal of Digital Literacy.

[CR14] Bozkurt A, Sharma R (2020). Emergency remote teaching in a time of global crisis due to CoronaVirus pandemic. Asian Journal of Distance Education.

[CR15] Cai Z, Fan X, Du J (2016). Gender and attitudes toward technology use: A meta-analysis. Computers & Education.

[CR16] Celik V, Yesilyurt E (2013). Attitudes to technology, perceived computer self-efficacy and computer anxiety as predictors of computer supported education. Computers & Education.

[CR17] Cha, E.-S., Kim, K., & Erlen, J. (2007). Translation of scales in cross-cultural research: issues and techniques. JAN: Research Methodology, 58(4), 386–395. 10.1111/j.1365-2648.2007.04242.x 17442038

[CR18] Chai CS, Koh JHL, Tsai C-C, Tan LLW (2011). Modeling primary school pre-service teachers’ Technological Pedagogical Content Knowledge (TPACK) for meaningful learning with information and communication technology (ICT). Computers & Education.

[CR19] Christensen R, Knezek G, Voogt J, Knezek G (2008). Self-report measures and findings for information technology attitudes and competencies. International handbook of information technology in primary and secondary education.

[CR20] Davis N, Eickelmann B, Zaka P (2013). Restructuring of educational systems in the digital age from a co-evolutionary perspective. Journal of Computer Assisted Learning.

[CR21] Dong Y, Xu C, Chai C, Zhai X (2020). Exploring the structural relationship among teachers’ technostress, technological pedagogical content knowledge (TPACK), computer self-efficacy and school support. Asia-Pacific Educational Research.

[CR22] Drossel B, Eickelmann B, Gerick J (2016). Predictors of teachers’ use of ICT in school – the relevance of school characteristics, teachers’ attitudes and teacher collaboration. Educational and Information Technologies.

[CR23] Eickelmann B (2011). Supportive and hindering factors to a sustainable implementation of ICT in schools. Journal for Educational Research Online/journal Für Bildungsforschung Online.

[CR24] Farjon D, Smits A, Voogt J (2019). Technology integration of pre-service teachers explained by attitudes and beliefs, competency, access and experience. Computers & Education.

[CR25] Ferrari A (2013). DIGCOMP: A framework for developing and understanding digital competence in Europe.

[CR26] Ferreira, E. (2017). The co-production of gender and ICT: Gender stereotypes in schools. *First Monday*, 22(10). 10.5210/fm.v22i10.7062

[CR27] Field A (2013). Discovering Statistics Using IBM SPSS Statistics.

[CR28] Fraillon, J., Ainley, J., Schulz,W., Friedman, T., & Duckworth, D. (Eds). (2019). *Preparing for life in a digital age: IEA international computer and information literacy study 2018 international report*. International Energy Agency.

[CR29] Fraillon J, Ainley J, Schulz W, Friedman T, Gebhardt E (2014). Preparing for life in a digital age.

[CR104] Gebhardt, E., Thomson, S., Ainley, J., & Hillman, K. (2019). *Gender differences in computer and information literacy. An In-depth analysis of data from ICILS*. Cham: Springer. 10.1007/978-3-030-26203-7_1.

[CR30] Gill L, Dalgarno B, Carlson L (2015). How Does Pre-Service Teacher Preparedness to Use ICTs for Learning and Teaching Develop Through Their Degree Program?. Australian Journal of Teacher Education.

[CR31] Gil-Flores J, Rodríguez-Santero J, Torres-Gordillo J-J (2017). Factors that explain the use of ICT in secondary-education classrooms: The role of teacher characteristics and school infrastructure. Computers in Human Behavior.

[CR32] Grafe, S., & Breiter, A. (2014). Modeling and measuring pedagogical media competencies of pre-service teachers (M^3^K). KoKoHs Working Papers from the BMBF-funded research initiative. https://www.blogs.uni-mainz.de/fb03-kokohs/files/2018/05/WP_No._6_Current_International_State_and_Future_Perspectives_on_Competence_Assessment_in_Higher_Education.pdf#page=80

[CR33] Gretter S, Yadav A (2018). What do preservice teachers think about teaching media literacy? An exploratory study using the theory of planned behaviour. Journal of Media Literacy Education.

[CR34] Guillén-Gámez, F., Lugones, A. & Mayorga-Fernández, M. J. (2019). ICT use by pre-service foreign languages teachers according to gender, age and motivation. *Cogent Education*, 6(1). 10.1080/2331186X.2019.1574693.

[CR35] Haddock G., & Maio G.R. (2014). Einstellungen [Attitudes]. In Jonas K., Stroebe W. and Hewstone M. (Eds), *Sozialpsychologie [Social Psychology]*. Heidelberg: Springer.

[CR100] Hämäläinen R, Nissinen K, Mannonen J, Lämsä J, Leino K, Taajamo M (2021). Understanding teaching professionals’ digital competence: What do PIAAC and TALIS reveal about technology-related skills, attitudes, and knowledge?. Computers in Human Behavior.

[CR36] Hatlevik IKR, Hatlevik OE (2018). Students’ evaluation of digital information: The role teachers play and factors that influence variability in teacher behaviour. Computers in Human Behavior.

[CR102] Hatlevik OE, Arnseth HC (2012). ICT, teaching and leadership: How do teachers experience the importance of ICT-supportiveschool leaders?. Nordic Journal of Digital Literacy.

[CR37] Hobbs R, Cabral N, Ebrahimi A, Yoon J, Al-Humaidan R (2011). Field-based teacher education in elementary media literacy as means to promote global understanding. Action in Teacher Education.

[CR38] International Society for Technology in Education. (2008). *The ISTE National Educational Technology Standards and Performance Indicators for Teachers (NETS-T)*.

[CR101] Jäger-Biela, D., Kaspar, K., & König, J. (2020). Lerngelegenheiten Zum Erwerb Von Digitalisierungsbezogenen Medienkompetenzen [Opportunities to learn digital media competences.].” In K. Kaspar, M. Becker-Mrotzek, S. Hofhues, J. König, and D. Schmeinck (Eds.), Bildung, schule, digitalisierung [Education, School, Digitalisation] (pp. 66–72). Münster: Waxmann.

[CR39] Kaufman K (2015). Information communication technology: Challenges & some prospects from preservice education to the classroom. Mid-Atlantic Education Review.

[CR40] Knezek G, Christensen R (2016). Extending the will, skill, tool model of technology integration: Adding pedagogy as a new model construct. Journal of Computing in Higher Education.

[CR41] Knezek, G., Christensen, R., & Fluke, R. (2003). Testing a will, skill, tool model of technology integration. In *Paper presented to the Annual Meeting of the American Educational Research Association (AERA)*. Chicago, IL.

[CR42] Knezek, G., Christensen, R., Hancock, R., & Shoho, A. (February 2000). Toward a structural model of technology integration. *Paper presented at the Hawaii Educational Research Association Annual Conference*, Honolulu, HI.

[CR43] Krause, M., Pietzner, V., Dori, Y. J., & Eilks, I. (2017). Differences and developments in attitudes and self-efficacy of prospective chemistry teachers concerning the use of ICT in education. EURASIA Journal of Mathematics Science and Technology Education. 10.12973/eurasia.2017.00935a

[CR44] König J, Jäger-Biela DJ, Glutsch N (2020). Adapting to Online Teaching During COVID-19 School Closure: Teacher Education and Teacher Competence Effects Early Career Teachers in Germany. European Journal of Teacher Education.

[CR45] Kreijns K, van Acker F, Vermeulen M, van Buuren H (2013). What stimulates teachers to integrate ICT in their pedagogical practices? The use of digital learning materials in education. Computers in Human Behavior.

[CR46] Maderick J, Zhang S, Hartley K, Marchand G (2015). Preservice teachers and self-assessing digital competence. Journal of Educational Computing.

[CR47] Markauskaite L (2006). Gender issues in preservice teachers’ training: ICT literacy and online learning. Australasian Journal of Educational Technology.

[CR48] Meelissen MR, Drent M (2008). Gender differences in computer attitudes: Does the school matter?. Computers in Human Behaviour.

[CR103] Morales, C. (2006). Cross-cultural validation of the will, skill, tool model of technology integration. Unpublished Doctoral Dissertation, University of North Texas, Denton, TX.

[CR49] Nistor, N., Wagner, M., & Heymann, J. O. (2012). Prädiktoren und Moderatoren der Akzeptanz von Bildungstechnologien. Die Unified Theory of Acceptance and Use of Technology auf dem Prüfstand [Predictors and moderators of technology acceptance: The unified Theory of Acceptance and use of technology in research]. In Zentrum für Empirische Pädagogische Forschung (Ed), *Empirische Pädagogik* [*Empirical Pedagogy*], 26(3), Landau in der Pfalz: Publisher Empirische Pädagogik.

[CR50] OECD. (2010). *Are the new millennium learners making the grade? Technology use and educational performance in PISA*. Derby: Centre for Educational Research and Innovation.

[CR51] OECD. (2014). *PISA 2012 results in focus. What 15*-*year*-*olds know and what they can do with what they know.* OECD: Paris.

[CR52] Pacurar E, Abbas N (2015). Analysis of French secondary school teachers’ intention to integrate digital work environments into their teaching practices. Education and Information Technologies.

[CR53] Petko D (2012). Teachers’ pedagogical beliefs and their use of digital media in classrooms: Sharpening the focus of the ‘will, skill, tool’ model and integrating teachers’ constructivist orientations. Computers & Education.

[CR54] Polly D, Mims C, Shepherd C, Inan F (2010). Evidence of impact: Transforming teacher education with preparing tomorrow’s teachers to teach with technology (PT3) grants. Teaching and Teacher Education.

[CR55] Rodrigues, S. (ed.). (2010). *Multiple literacy and science education: ICTs in formal and informal learning environments.* Hershey: IGI Global.

[CR56] Rubach, C., & Lazarides, R. (2019). Eine Skala zur Selbsteinschätzung digitaler Kompetenzen bei Lehramtsstudierenden. *Zeitschrift für Bildungsforschung [Journal for Educational Research]*. 10.1007/s35834-019-00248-0

[CR57] Rubach C, Lazarides R (2020). Digitale Kompetenzeinschätzungen von Lehramtsstudierenden fördern [Pre-service teachers self-assessment of their digital competences]. Journal Für LehrerInnenbildung [journal for Teacher Education].

[CR58] Rubach C, Lazarides R (2021). Addressing 21st-centura digital skills in schools – Development and validation of an instrument to measure teachers’ basic ICT competence beliefs. Computers in Human Behavior.

[CR59] Sá, M. J., & Serpa, S. (2020). The global crisis brought about by SARS-CoV-2 and its impacts on education: An overview of the Portuguese panorama. *Scientific Insight Educational Frontiers*, 5(2), 525–530. 525–530. http://dx.doi.org/10.2139/ssrn.3565613

[CR60] Sang G, Valcke M, van Braak J, Tondeur J (2010). Student teachers’ thinking processes and ICT integration: Predictors of prospective teaching behaviors with educational technology. Computers & Education.

[CR61] Savin NE, White KJ (1978). Testing for autocorrelation with missing observations. Econometrica.

[CR62] Schaarschmidt U, Schaarschmidt U (2005). Potsdamer Lehrerstudie – ein erstes Fazit [Potsdam Teacher Study: First results]. Halbtagsjobber? Psychische Gesundheit im Lehrerberuf – Analyse eines veränderungsbedürftigen Zustands [Part-time Job? Mental health in the teaching profession – an analysis of the changing necessities].

[CR63] Scherer R, Siddiq F (2015). Revisiting teachers’ computer self-efficacy: A differentiated view on gender differences. Computers in Human Behavior.

[CR64] Scherer R, Teo T (2019). Unpacking teachers‘ intentions to integrate technology: A meta-analysis. Educational Research Review.

[CR65] Schuknecht, L., & Schleicher, A. (2020). Digital Herausforderungen für Schulen und Bildung [Digital challenges for schools and education]. *ifo Schnelldienst, ifo Institute - Leibniz Institute for Economic Research at the University of Munich*, 73(5), 68–70.

[CR66] Seufert S, Guggemos J, Sailer M (2021). Technology-related knowledge, skills and attitudes of pre- and in-service teachers: The current situation and emerging trends. Computers in Human Behaviour.

[CR67] Siddiq F, Scherer R, Tondeur J (2015). Teachers’ Emphasis on Developing Students’ Digital Information and Communication Skills (TEDDICS): A New Construct in 21st Century Education. Computers & Education.

[CR68] Spiteri M, Rundgren S-N (2020). Literature review on the factors affecting primary teachers’ use of digital technology. Technology, Knowledge and Learning..

[CR69] Stephan M, Markus S, Gläser-Zikuda M (2019). Students’ Achievement Emotions and Online Learning in Teacher Education. Frontiers in Education.

[CR70] Shulman LS (1986). Those who understand: Knowledge growth in teaching. Educational Researcher.

[CR71] Tappe, E.-H. (2017). *Lernen durch Mediengestaltung – Entwicklung eines Konzeptes zur Unterstützung mediendidaktischer Lehre im Schulalltag [Learning through media lessons – Development of a concept to support teaching with media in school].* Doctoral dissertation, Wilhelms University in Münster. https://d-nb.info/1163319627/34

[CR72] Teo T (2008). Pre-service teachers’ attitudes towards computer use: A Singapore survey. Australasian Journal of Educational Technology.

[CR73] The Standing Conference of Ministers of Education and Culture (KMK). (2016). *Education in the Digital World*. https://www.kmk.org/fileadmin/Dateien/pdf/PresseUndAktuelles/2017/KMK-Strategie_Bildung_in_der_digitalen_Welt_Zusammenfassung_en.pdf

[CR74] Tiede, J. (2020). Conclusions: Media-related educational competencies of German and US preservice teachers. *MedienPädagogik*, 221–235.

[CR75] Tiede J, Grafe S, Hobbs R (2015). Pedagogical media competencies of preservice teachers in Germany and the United States: A comparative analysis of theory and practice. Peabody Journal of Education.

[CR76] Tømte, C. (2008). *‘Return to gender’: Gender, ICT and education. Background paper of OECD Expert meeting hosted by the Norwegian Ministry of Education and Research*. Paris: OECD, at http://www.oecd.org/edu/ceri/40834253.pdf, accessed 07 June 2021.

[CR77] Tondeur J, Aesaert K, Prestridge S, Consuegra E (2018). A multilevel analysis of what matters in the training of pre-service teacher's ICT competencies. Computers & Education.

[CR78] Tondeur, J., Aesaert, K., Pynoo, B., van Braak, J., Fraeyman, N., & Erstad, O. (2017). Developing a validated instrument to measure preservice teachers’ ICT competencies: Meeting the demands of the 21st century. *British Journal of Educational Technology*, 48(2). 10.1111/bjet.12380

[CR79] Tondeur J, Kershaw LH, Vanderlinde R, Van Braak J (2013). Getting inside the black boy of technology integration in education: Teachers’ simulated recall of classroom observations. Australasian Journal of Educational Technology.

[CR80] Tondeur J, Petko D, Christensen R, Drossel K, Starkey L, Knezek; G., & Schmidt-Crawford, D.  (2020). Quality criteria for conceptual technology integration models in education: Bridging research and practice. Educational Technology Research and Development.

[CR81] Tondeur J, van Braak J, Sang G, Voogt J, Fisser P, Ottenbreit-Leftwich A (2012). Preparing preservice teachers to integrate technology in education: A synthesis of qualitative evidence. Computers & Education.

[CR82] Tondeur, J., Van de Velde, S., Vermeersch, H. & Van Houtte, M. (2016). Gender Differences in the ICT Profile of University Students: A Quantitative Analysis. *DiGeSt. Journal of Diversity and Gender Studies*, 3(1), 57–77. 10.11116/jdivegendstud.3.1.0057

[CR83] Tondeur J, van Keer H, van Braak J, Valcke M (2008). ICT integration in the classroom: Challenging the potential of a school policy. Computers & Education.

[CR84] Valtonen, T., Hoang, N., Sointu, E., Näykki, P. Virtanen, A., Pöysa-Tarhonen, J. et al. (2021). How pre-service teachers perceive their 21^st^-century skills and dispositions: A longitudinal perspective. *Computers in Human Behavior*, *116*.

[CR85] UNESCO (2011). UNESCO ICT Competency Framework for Teachers.

[CR86] Urezm D, Volman M, Kral M (2018). Teacher educators’ competences in fostering student teachers’ proficiency in teaching and learning with technology: An overview of relevant research literature. Teacher and Teacher Education.

[CR87] Woolfolk A (2004). Educational psychology.

